# A Self-Stabilized Inverted Pendulum Made of Optically Responsive Liquid Crystal Elastomers

**DOI:** 10.3389/frobt.2021.808262

**Published:** 2022-01-11

**Authors:** Quanbao Cheng, Lin Zhou, Kai Li

**Affiliations:** ^1^ Department of Civil Engineering, Anhui Jianzhu University, Hefei, China; ^2^ School of Mechanical and Electrical Engineering, Anhui Jianzhu University, Hefei, China

**Keywords:** inverted pendulum, stabilization, liquid crystal elastomer, optically-responsive, self-sustained motion

## Abstract

The inverted pendulum system has great potential for various engineering applications, and its stabilization is challenging because of its unstable characteristic. The well-known Kapitza’s pendulum adopts the parametrically excited oscillation to stabilize itself, which generally requires a complex controller. In this paper, self-sustained oscillation is utilized to stabilize an inverted pendulum, which is made of a V-shaped, optically responsive liquid crystal elastomer (LCE) bar under steady illumination. Based on the well-established dynamic LCE model, a theoretical model of the LCE inverted pendulum is formulated, and numerical calculations show that it always develops into the unstable static state or the self-stabilized oscillation state. The mechanism of the self-stabilized oscillation originates from the reversal of the gravity moment of the inverted pendulum accompanied with its own movement. The critical condition for triggering self-stabilized oscillation is fully investigated, and the effects of the system parameters on the stability of the inverted pendulum are explored. The self-stabilized inverted pendulum does not need an additional controller and offers new designs of self-stabilized inverted pendulum systems for potential applications in robotics, military industry, aerospace, and other fields.

## I. Introduction

The pendulum in an inverted position is a multivariate, high-order, nonlinear, strong coupling, and natural unstable system (Nivedita and Soumitro, (2020); Gonzalez and Rossiter, (2020)). The implementation of an inverted pendulum system can effectively reflect many typical problems in control, such as nonlinear, robustness, stabilization, follow-up, and tracking problems (Junkun et al. (2020); Atilla and Firat, (2020)). The stabilization of the inverted pendulum can be used to test whether a new control method has a strong ability to deal with nonlinear and unstable problems. At the same time, its stabilization methods are widely used in the fields of robotics, military industry, aerospace, and other fields, such as stabilization in the robot walking process, perpendicularity control in rocket launch, and attitude control in satellite flight (Balcerzak (2020)). The challenge of studying the inverted pendulum system is not only the control difficulty caused by the multistage inverted pendulum, but also its own complexity, instability, and nonlinear characteristics. Therefore, it is required to study and expand new theoretical methods to apply to new control objects and provide a better experimental theory and platform (Zheng et al. (2020)).

At present, various inverted pendulum systems are proposed, in which a complex controller is usually required to achieve its stabilization. For example, the well-known Kapitza’s pendulum adopts parametrically excited oscillation to stabilize itself, which requires additional controllers to apply specific periodic external forces (Kapitza (1951)). To simplify the system, self-excited oscillation may be used to achieve self-stabilization of inverted pendulums (Li et al. (2003); Maeda et al. (2007); Serak et al. (2010); Jenkins (2013)). Self-excited oscillation is a phenomenon in which the system has continuous state change under constant external stimulation (Uchida et al. (2015); Kumar et al. (2016); Nocentini et al. (2018)). Various self-excited oscillations are constructed based on many kinds of passive and active material systems (Kinoshita (2013); Chakrabarti et al. (2020); Bartlett et al. (2015); Wehner et al. (2016)). Because of their unique advantages, self-excited oscillations have broad application prospects in many fields, such as energy harvesters (Baumann et al. (2018)), soft robots (Nocentini et al. (2018)), medical devices (Hu et al. (2018)), and micro/nano devices (Huang and Aida, (2019)). The stimuli-responsive materials for the self-excited oscillation systems include hydrogels, ionic gels, liquid crystal elastomer (LCE), etc. Different from classic conservative systems, the energy loss of self-excited oscillation caused by system damping requires external energy inflow and energy compensation. Based on different stimuli-responsive materials and structures, different feedback mechanisms are proposed to realize energy compensation, such as a coupling mechanism between chemical reaction (Lahikainen et al., 2018) and large deformation (Cheng et al., 2019), a self-shading mechanism (Serak et al., 2010), and a coupling mechanism in a droplet evaporation multiprocess (Chakrabarti et al., 2020). These mechanisms originate from the nonlinear coupling of multiple processes for implementing feedback.

Light is an excitation with the unique advantages of remote precise control, no noise, being clean, and so on (Kim et al. (2021); Adam et al. (2018)). In addition, these advantages of light make it more convenient to induce customized feedback to realize self-excited oscillation by various means. LCE is a polymer network structure formed by crosslinking liquid crystal monomer molecules (Gelebart et al. (2017)). When stimulated by external fields, such as light, heat, electricity, and magnetism, liquid crystal monomer molecules rotate or undergo phase transition to change their configurations, which induce macroscopic deformation (Boissonade and Kepper, (2011); Li et al. (2014)). LCE generally has the characteristics of rapid deformation response, deformation recovery, and being noiseless. Compared with other types of active materials, LCE also has several unique advantages (Lee et al. (2011); Yamada et al. (2008); Cheng et al. (2021)). For example, compared with pneumatic artificial muscles, temperature-sensitive gels, and moisture-sensitive gels, optically responsive LCE has the advantage of wireless, contactless driving, which is conducive to a lightweight structure and less affected by the environment. Moreover, different from polyelectrolyte gels, there are no chemical by-products for optically responsive LCE.

Based on a light-fueled, self-excited oscillation, we propose a new self-stabilized inverted pendulum system in this paper. It is made up of a V-shaped LCE bar and can autonomously rotate around its pivot under steady illumination. The inverted pendulum does not need an additional controller, which simplifies the system and provides new designs of self-stabilized inverted pendulum systems for potential applications. The object of the paper is to theoretically study the self-stabilization of the LCE inverted pendulum under steady illumination, elucidate the mechanism of the self-stabilized oscillation, and systematically investigate the effects of various physical and geometric parameters on the motion modes, its amplitude, and period. The text reads as follows. In Sec. II, based on the well-established dynamic LCE model, the governing equation of the LCE inverted pendulum is derived, and then the difference scheme and solution method for the dynamic equations are given. In Sec. III, the two motion modes of the system are discussed, and the detailed mechanisms are elucidated. In Sec. IV, parameter analysis is carried out to investigate the influence of various parameters on the triggering condition, amplitude, and period of the self-stabilized oscillation. The final section presents the conclusion.

## II. Model and Formulation

### A. Dynamics of the LCE Inverted Pendulum

**FIGURE 1:**
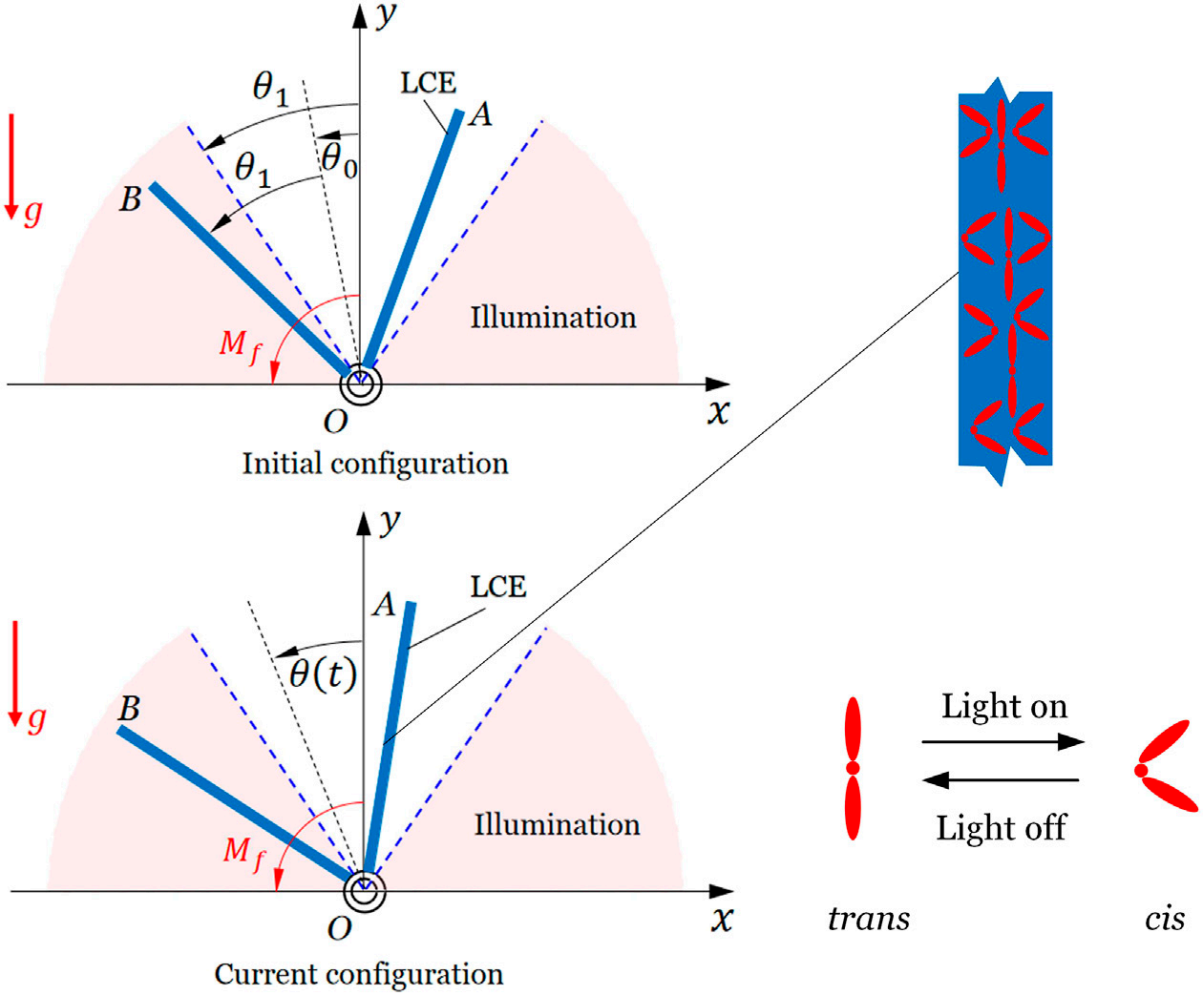
Schematic of an inverted pendulum made up of a V-shaped optically responsive LCE bar. Under steady illumination, the inverted pendulum can be self-stabilized due to the reversal of gravity moment resulting from light-driven contraction of the LCE bars.

Figure 1 sketches an inverted pendulum made up of a V-shaped optically responsive LCE bar, which can be constructed by rigidly connecting two LCE bars fabricated by the two-step method (Yakacki et al. (2015)). The two bars, OA and OB, have the same length, width, and thickness and have the same mass m . The V-shaped LCE bar is in the xOy plane and can rotate about its pivot O . The vertex angle of the V-shaped LCE bar is $2\theta_{1}$ , and the nonillumination zone is set to be $\left(-\theta_{1},\theta_{1}\right)$ . There may be a critical value between 0° and 90°, which is discussed in detail in section IV. The original length of the two bars before being illuminated is $l_{0}$ . The initial position of the V-shaped LCE bar is denoted by the angle $\theta_{0}$ between its symmetry axis and y -axis, and the initial angular velocity is set to be zero. The current position is denoted by the angle θ(t) . We assume that the thickness of the V-shaped LCE bar is much smaller than the penetration depth of the light. Therefore, the two bars under illumination only contract, and their lengths $l_{A}\left(t\right)$ and $l_{B}\left(t\right)$ vary with time, which causes the change of the gravity moment and, in turn, results in self-stabilized oscillation of the LCE inverted pendulum.

According to angular momentum theorem, dynamics of the LCE inverted pendulum are governed by

$$\frac{d\Phi }{dt}=M_{Z},$$ (1)

where the angular momentum Φ of the inverted pendulum about the pivot is

$$\Phi =J_{z}\left(t\right)\frac{d\theta \left(t\right)}{dt},$$ (2)

where $J_{\text{z}}$ is the moment of inertia of the V-shaped LCE bar about the pivot,

$$J_{z}=J_{z1}+J_{z2},$$ (3)

where $J_{z1}=\frac{1}{3}ml_{A}^{2}$ is the moment of inertia of OA about the pivot, and $J_{z2}=\frac{1}{3}ml_{B}^{2}$ is the moment of inertia of OB about the pivot. During the formulation above, the rod is assumed to be rigid, and the bending under the applied torques is ignored for the ratio of the deflection to the length is estimated to be very small for the typical value of the parameters given in Sec. III.

The current lengths of OA and OB bars are

$$l_{A}=\left[1+\varepsilon_{1}\left(t\right)\right]l_{0},\;l_{B}=\left[1+\varepsilon_{2}\left(t\right)\right]l_{0},$$ (4)

where $\varepsilon_{1}\left(t\right)$ and $\varepsilon_{2}\left(t\right)$ are the light-driven contraction strain of OA and OB, respectively. For simplicity, the light-driven contraction strain of the material is assumed to be proportional to the *cis* number fraction φ(t) ,

$$\varepsilon_{1}\left(t\right)=-C_{0}\varphi_{1}\left(t\right),\varepsilon_{2}\left(t\right)=-C_{0}\varphi_{2}\left(t\right),$$ (5)

where $C_{0}$ is the contraction coefficient. The number fraction ϕ(t) is given in the following section IIB.

In Eq. 1, $M_{z}$ is the total moment of all the external forces about its pivot. The damping moment is assumed to be proportional to the angular velocity. Therefore, the total moment is given as

$$M_{z}=M_{g}-M_{f},$$ (6)

where the gravity moment $M_{g}=\frac{1}{2}mg\left[l_{B}\sin\left(\theta_{1}+\theta \right)-l_{A}\sin\left(\theta_{1}-\theta \right)\right]$ , g is the gravitational acceleration, the damping moment $M_{f}=\frac{1}{3}\zeta \left(l_{A}^{3}+l_{B}^{3}\right)\frac{d\theta }{dt}$ , ζ is the damping coefficient, and $\frac{d\theta }{dt}=\dot{\theta }$ is the angular velocity of the inverted pendulum.

### B. Dynamic LCE Model

To determine the motion of the inverted pendulum, we first obtain the current lengths of OA and OB, which depend on the number fraction of *cis* isomers in the LCE bars. Here, the well-established dynamic LCE model is utilized to determine the number fraction (Warner and Terentjev, (2003)). Generally, the number fraction of *cis* isomers depends on thermal excitation from *trans* to *cis*, thermally driven relaxation from *cis* to *trans*, and light-driven *trans*-to-*cis* isomerization. Considering that thermal excitation from *trans* to *cis* is often negligible relative to the light-driven excitation, the evolution of the number fraction of bent *cis* isomers is derived as (Warner and Terentjev, (2003)),

$$\frac{\partial \varphi }{\partial t}=\eta_{0}I_{0}\left(1-\varphi \right)-\frac{\varphi }{T_{0}},$$ (7)

where $\eta_{0}$ is the light absorption constant, $I_{0}$ is the light intensity, and $T_{0}$ is the thermal relaxation time from *cis* to *trans* state. The solution to Eq. 7 can be easily obtained as

$$\varphi \left(t\right)=\frac{\eta_{0}T_{0}I_{0}}{\eta_{0}T_{0}I_{0}+1}+\left(\varphi_{0}-\frac{\eta_{0}T_{0}I_{0}}{\eta_{0}T_{0}I_{0}+1}\right)\exp\left[-\frac{t}{T_{0}}\left(\eta_{0}T_{0}I_{0}+1\right)\right],$$ (8)

where $\varphi_{0}$ is the number fraction of *cis* isomers at t=0 . In the light zone, for initially zero number fraction of *cis* isomers, i.e., $\varphi_{0}=0$ , Eq. 8 can be simplified as

$$\varphi \left(t\right)=\frac{\eta_{0}T_{0}I_{0}}{\eta_{0}T_{0}I_{0}+1}\left\{1-\exp\left[-\frac{t}{T_{0}}\left(1+\eta_{0}T_{0}I_{0}\right)\right]\right\}.$$ (9)

In the dark zone, namely, $I_{0}=0$ , $\varphi_{0}$ can be set as the maximum of φ(t) in Eq. 9, namely, $\varphi_{0}=\frac{\eta_{0}T_{0}I_{0}}{\eta_{0}T_{0}I_{0}+1}$ , and Eq. 8 can be simplified as

$$\varphi \left(t\right)=\frac{\eta_{0}T_{0}I_{0}}{\eta_{0}T_{0}I_{0}+1}\exp\left(-\frac{t}{T_{0}}\right).$$ (10)

By defining the dimensionless quantities $\bar{I}=\eta_{0}T_{0}I_{0}$ , $\bar{t}=t/T_{0}$ and $\bar{\varphi }=\varphi \left(\eta_{0}T_{0}I_{0}+1\right)/\eta_{0}T_{0}I_{0}$ in the light zone, Eq. 9 is rewritten as

$$\bar{\varphi }=1-\exp\left[-\bar{t}\left(\bar{I}+1\right)\right],$$ (11)

and in the dark zone, Eq. 10 is rewritten as

$$\bar{\varphi }=\exp\left(-\bar{t}\right).$$ (12)

### C. Governing Equations of the LCE Inverted Pendulum

Here, we define the following dimensionless quantities: $\bar{g}=gT_{0}^{2}/l_{0}$ , $\bar{\zeta }=2\zeta T_{0}l_{0}/m$ , and ${\bar{M}}_{D}=2M_{D}T_{0}^{2}/\left(ml_{0}^{2}\right)$ . It is noteworthy that $\bar{g}$ can be rewritten as $\bar{g}={\left(T_{0}/\sqrt{l_{0}/g}\right)}^{2}$ by the natural period $\sqrt{l_{0}/g}$ of a single pendulum, which represents the *cis*-to-*trans* thermal relaxation time relative to the natural period. The larger $\bar{g}$ is, the slower the *cis*-to-*trans* conversion.

Considering that the width is much smaller than the amplitude of oscillation, and the time of the instantaneous transition between bright and dark is also much smaller than a period, the transition time is ignored in the computation. Combining Eqs 2–5, 11, 12 leads to for θ≥0 (i.e., only the OB bar is illuminated),

$$\frac{d^{2}\theta \left(\bar{t}\right)}{d{\bar{t}}^{2}}=A_{1}\left(\bar{t}\right)\frac{d\theta \left(\bar{t}\right)}{d\bar{t}}+B_{1}\left(\bar{t}\right),$$ (13)

where,

$$A_{1}\left(\bar{t}\right)=\frac{-4C_{0}\bar{I}\exp\left(-\bar{t}\right)\left[\left(1+\varepsilon_{1}\right)/\left(\bar{I}+1\right)-\left(1+\varepsilon_{2}\right)\exp\left(-\bar{I}\bar{t}\right)\right]-\bar{\zeta }\left[{\left(1+\varepsilon_{1}\right)}^{3}+{\left(1+\varepsilon_{2}\right)}^{3}\right]}{2{\left(1+\varepsilon_{1}\right)}^{2}+2{\left(1+\varepsilon_{2}\right)}^{2}}$$,

$$B_{1}\left(\bar{t}\right)=\frac{3\bar{g}\left\{\left[1+\varepsilon_{2}\left(\bar{t}\right)\right]\sin\left(\theta_{1}+\theta \right)-\left[1+\varepsilon_{1}\left(\bar{t}\right)\right]\sin\left(\theta_{1}-\theta \right)\right\}}{2{\left(1+\varepsilon_{1}\right)}^{2}+2{\left(1+\varepsilon_{2}\right)}^{2}}$$

$\varepsilon_{1}=-C_{0}\bar{I}\exp\left(-\bar{t}\right)/\left(\bar{I}+1\right)$ and $\varepsilon_{2}=-C_{0}\bar{I}\left\{1-\exp\left[-\bar{t}\left(\bar{I}+1\right)\right]\right\}/\left(\bar{I}+1\right)$ , and for θ<0 (i.e., only the OA bar is illuminated),

$$\frac{d^{2}\theta \left(\bar{t}\right)}{d{\bar{t}}^{2}}=A_{2}\left(\bar{t}\right)\frac{d\theta \left(\bar{t}\right)}{d\bar{t}}+B_{2}\left(\bar{t}\right),$$ (14)

where,

$$A_{2}\left(\bar{t}\right)=\frac{-4C_{0}\bar{I}\exp\left(-\bar{t}\right)\left[\left(1+\varepsilon_{2}\right)/\left(\bar{I}+1\right)-\left(1+\varepsilon_{1}\right)\exp\left(-\bar{I}\bar{t}\right)\right]-\bar{\zeta }\left[{\left(1+\varepsilon_{1}\right)}^{3}+{\left(1+\varepsilon_{2}\right)}^{3}\right]}{2{\left(1+\varepsilon_{1}\right)}^{2}+2{\left(1+\varepsilon_{2}\right)}^{2}}$$,

$$B_{2}\left(\bar{t}\right)=\frac{3\bar{g}\left\{\left[1+\varepsilon_{2}\left(\bar{t}\right)\right]\sin\left(\theta_{1}+\theta \right)-\left[1+\varepsilon_{1}\left(\bar{t}\right)\right]\sin\left(\theta_{1}-\theta \right)\right\}}{2{\left(1+\varepsilon_{1}\right)}^{2}+2{\left(1+\varepsilon_{2}\right)}^{2}}$$

$\varepsilon_{1}=-C_{0}\bar{I}\left\{1-\exp\left[-\bar{t}\left(\bar{I}+1\right)\right]\right\}/\left(\bar{I}+1\right)$ and $\varepsilon_{2}=-C_{0}\bar{I}\exp\left(-\bar{t}\right)/\left(\bar{I}+1\right)$ .

### D. Solution Method

Equations 13, 14 are ordinary differential equations with variable coefficients, and there exists no analytic solution. Hereon, the classic fourth order Runge–Kutta method is used to numerically solve the ordinary differential equations by software *Matlab*. We first transform the second order ordinary differential equation with variable coefficients into two first order ordinary differential equations with variable coefficients. Therefore, the governing equations are rewritten as

$$\{\begin{array}{c} \frac{d\theta \left(\bar{t}\right)}{d\bar{t}}=\dot{\theta }\\ \frac{d^{2}\theta }{d{\bar{t}}^{2}}=f\left(\bar{t},\theta ,\dot{\theta }\right)\\ \dot{\theta }\left(\bar{t}=0\right)={\dot{\theta }}_{0}\\ \theta \left(\bar{t}=0\right)=\theta_{0} \end{array},$$ (15)

where

$$f\left(\bar{t},\theta ,\dot{\theta }\right)=\left\{ \begin{array}{l} A_{1}\left(\bar{t}\right)\frac{d\theta \left(\bar{t}\right)}{d\bar{t}}+B_{1}\left(\bar{t}\right),for\;\;\;\theta \geq 0\\ A_{2}\left(\bar{t}\right)\frac{d\theta \left(\bar{t}\right)}{d\bar{t}}+B_{2}\left(\bar{t}\right),for\;\;\;\theta <0 \end{array}\right..$$ (16)

The classic fourth order Runge–Kutta method is used to solve the problem, The final steady-state of the inverted pendulum is obtained by iteration. When the bar switches between light on and light off, the evolution law is correspondingly converted between Eqs 11, 12. At the conversion moment, the cis number fraction ϕ(t) is kept unchanged. On the basis of this, the new time point can be acquired when the state switches. For example, when the bar rotates from the light on region to the light off region, the time conversion law is ${\bar{t}}_{\mathrm{off}}=-\ln\left\{1-\exp\left[-{\bar{t}}_{\mathrm{on}}\left(\bar{I}+1\right)\right]\right\}$ . Numerically, the time point ${\bar{t}}_{\mathrm{off}}$ in the light off region can be computed by means of ${\bar{t}}_{\mathrm{on}}$ in the light on region. In the light off region, the subsequent motion begins with the time point ${\bar{t}}_{\mathrm{off}}$ . On the contrary, when the bar rotates from the dark to the light region, the time conversion rule is ${\bar{t}}_{\mathrm{on}}=-\ln\left[1-\exp\left(-{\bar{t}}_{\mathrm{off}}\right)\right]/\left(\bar{I}+1\right)$ . The time point ${\bar{t}}_{\mathrm{on}}$ in the light on region can be computed numerically in terms of ${\bar{t}}_{\mathrm{off}}$ in the light off region. In the light on region, the following motion begins with the time point ${\bar{t}}_{\mathrm{on}}$ .

## III. Two Motion Modes and Mechanisms

After numerical computations with respect to Eqs 13, 14 in Sec. IIC, a series of results can be obtained with the variation of physical parameters related to oscillation. In numerical calculations, we choose the typical values of physical parameters from accessible experiments (Marshall and Terentjev, (2013); Nagele et al. (1997)) as follows: l=5mm , b=0.5mm , h=0.5mm , $\rho =10^{3}\mathrm{Kg}/m^{3}$ , E=1MPa , $C_{0}=0.5$ , I=0105W⋅m-2 , $T_{0}=10^{-1}s$ , $\eta_{0}=10^{-4}$ . Then, the corresponding dimensionless parameters are estimated as $\bar{I}=0∼1$ , $\bar{g}=1∼10^{2}$ , $\bar{\zeta }=0∼10^{2}$ . The numerical calculations show that the initial condition of the number fraction does not affect the motion mode. For simplicity, in Eqs 11, 12 , we assume that initially the OA bar is in the nonilluminated state of $\bar{\phi }=0$ , whereas the OB bar is in a fully illuminated state of $\bar{\phi }=1$ , which is easily achieved experimentally.

### A. Self-Stabilized Oscillation of the LCE Inverted Pendulum

**FIGURE 2:**
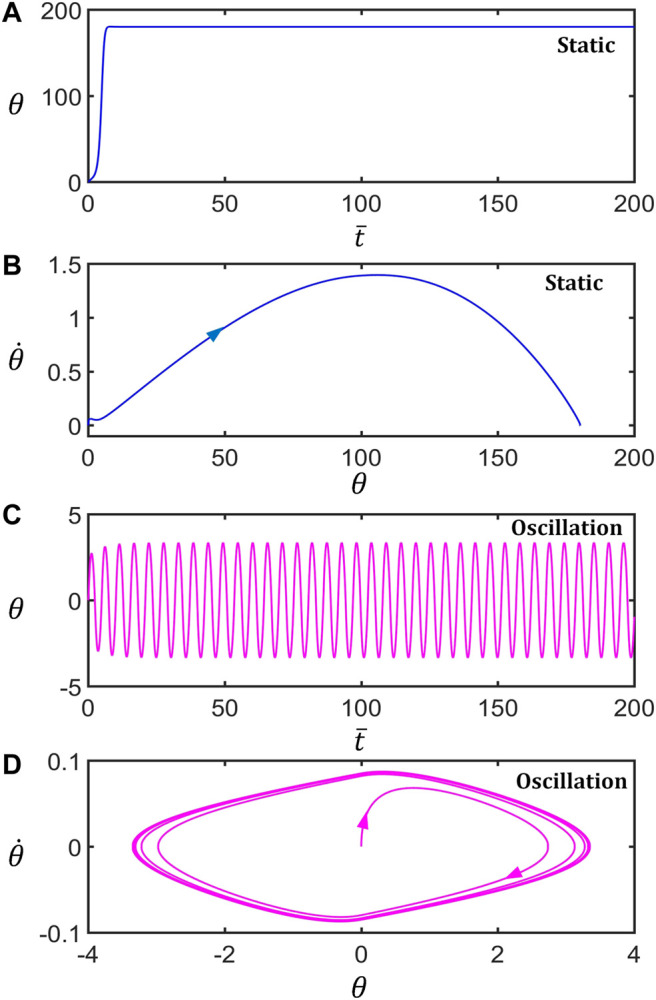
Two motion modes of the light-powered inverted pendulum. **(A)** and **(C)** are the time series curves of the unstable static mode $\left(\theta_{1}=50{}^{\circ}\right)$ and self-stabilized oscillation mode $\left(\theta_{1}=70{}^{\circ}\right)$ , respectively. **(B)** and **(D)** are phase diagrams of the unstable static mode and self-stabilized oscillation mode, respectively. The other parameters are $\bar{I}=0.25$ , $C_{0}=0.4$ , $\bar{g}=9.8$ , $\bar{\zeta }=14.7$ , $\theta_{0}=0{}^{\circ}$ , and ${\dot{\theta }}_{0}=0$ .

Figure 2 shows two typical motion modes of the LCE inverted pendulum: the unstable static mode and the self-stabilized oscillation mode. Figures 2A,B, respectively, draw the time series curve and its phase diagram of the rotation angle of the typical static mode for $\bar{I}=0.25$ , $C_{0}=0.4$ , $\bar{g}=9.8$ , $\bar{\zeta }=14.7$ , $\theta_{0}=0{}^{\circ}$ , $\theta_{1}=50{}^{\circ}$ , ${\dot{\theta }}_{0}=0$ . The results show that the inverted pendulum tilts and quickly comes to rest, reaching its lowest point and finally staying at a fixed point on the phase diagram. Figures 2C,D, respectively, draw the time series curve and its phase diagram of the rotation angle of the typical oscillation mode for $\bar{I}=0.25$ , $C_{0}=0.4$ , $\bar{g}=9.8$ , $\bar{\zeta }=14.7$ , $\theta_{0}=0{}^{\circ}$ , $\theta_{1}=70{}^{\circ}$ and ${\dot{\theta }}_{0}=0$ . The results show that the swing amplitude of the bar gradually becomes stable, and its state finally stays on the limit cycle in the phase diagram. In the following, we elucidate the mechanism of the self-stabilized oscillation.

### B. Mechanisms of the Self-Stabilized Oscillation

**FIGURE 3:**
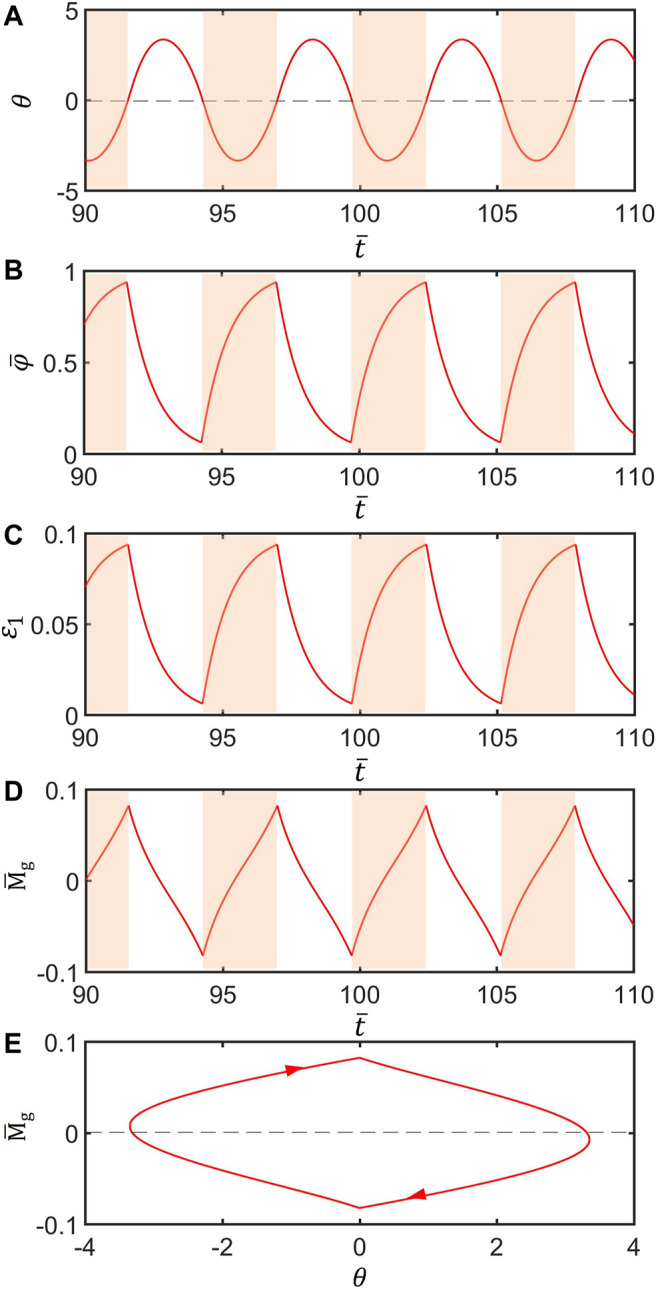
Mechanism of the self-stabilized oscillation of the LCE inverted pendulum. Parameters are set: $\bar{I}=0.25$ , $C_{0}=0.4$ , $\bar{g}=9.8$ , $\bar{\zeta }=14.7$ , $\theta_{0}=0{}^{\circ}$ , $\theta_{1}=70{}^{\circ}$ , and ${\dot{\theta }}_{0}=0$ . **(A)** The rotation angle as a function of time. **(B)** The variation of the number fraction of *cis*-isomers in the OA bar with time. **(C)** The contraction strain as a function of time. **(D)** Dependence of the gravity moment on time. **(E)** The variation of the gravity moment with the angle of rotation.

To study how the LCE inverted pendulum compensates for the damping dissipation to maintain the periodic oscillation, the time series curves of each physical quantity in a typical mode of the inverted pendulum are given in Figure 3. Parameters are set as follows: $\bar{I}=0.25$ , $C_{0}=0.4$ , $\bar{g}=9.8$ , $\bar{\zeta }=14.7$ , $\theta_{0}=0{}^{\circ}$ , $\theta_{1}=70{}^{\circ}$ and ${\dot{\theta }}_{0}=0$ . Figures 3A,B describe the variation of the rotation angle and present the curve of the number fraction of *cis* isomers in the OA bar in the inverted pendulum system. The dark color indicates that θ<0 . For θ>0 , the number fraction of *cis* isomers of the OA bar gradually decreases although, for θ<0 , the number fraction of *cis* isomers of the OA bar gradually increases. Finally, the number fraction changes periodically. Figure 3C plots the variation of the strain in the OA bar with time. For θ>0 , the strain in the OA bar decreases gradually, and for θ<0 , the strain in the OA bar increases gradually. Similarly, the light-driven contraction changes periodically. Figure 3D plots the variation of the gravity moment of the system with time. For θ>0 , the gravity moment of the system decreases gradually, and for θ<0 , the gravity moment of the system increases gradually. The gravity moment also varies periodically. Figure 3E plots the dependence of the gravity moment on the angle in one cycle of the steady oscillation. In Figure 3E, the area surrounded by the closed curve represents the net work done by the light illumination, which compensates for the energy loss caused by damping to maintain the periodic oscillation of the system. This phenomenon can also be understood from the perspective of energy. In a cycle, the energy converted by the V-shaped inverted pendulum under light illumination is equal to the energy dissipated by the damping, and thus, the stable oscillation of the system can be maintained.

The inverted pendulum in this paper is stabilized through self-excited oscillation and is much different from the well-known Kapitza’s pendulum stabilized through parametrically excited oscillations (Kapitza (1951)). Generally, parametrically excited oscillations arise from the external excitation of periodic or system parameters and usually adjust the parameters of the oscillatory system through a clear process-independent time law. The control differential equations for parametrically excited oscillations generally have periodic time-varying coefficients. However, compared with parametrically excited oscillations, self-excited oscillations are caused by the internal interaction of the elements within the system in external constant energy situations, and the system can maintain the periodic motion of equal amplitude through process-related self-regulation and feedback control (Ding (2010)).

## IV. Parametric Study

Generally, the system oscillates with a limit cycle around a naturally unstable upper position due to the material properties. Considering the theoretical analysis of the bifurcation is very difficult, alternatively, we perform detailed numerical analysis to obtain the critical condition for triggering self-excited oscillation of the pendulum. Furthermore, we also investigate the effects of the system parameters on the amplitude and period of the self-stabilized oscillation of the inverted pendulum through the variable-controlling method.

### A. Effect of the Vertex Angle

**FIGURE 4:**
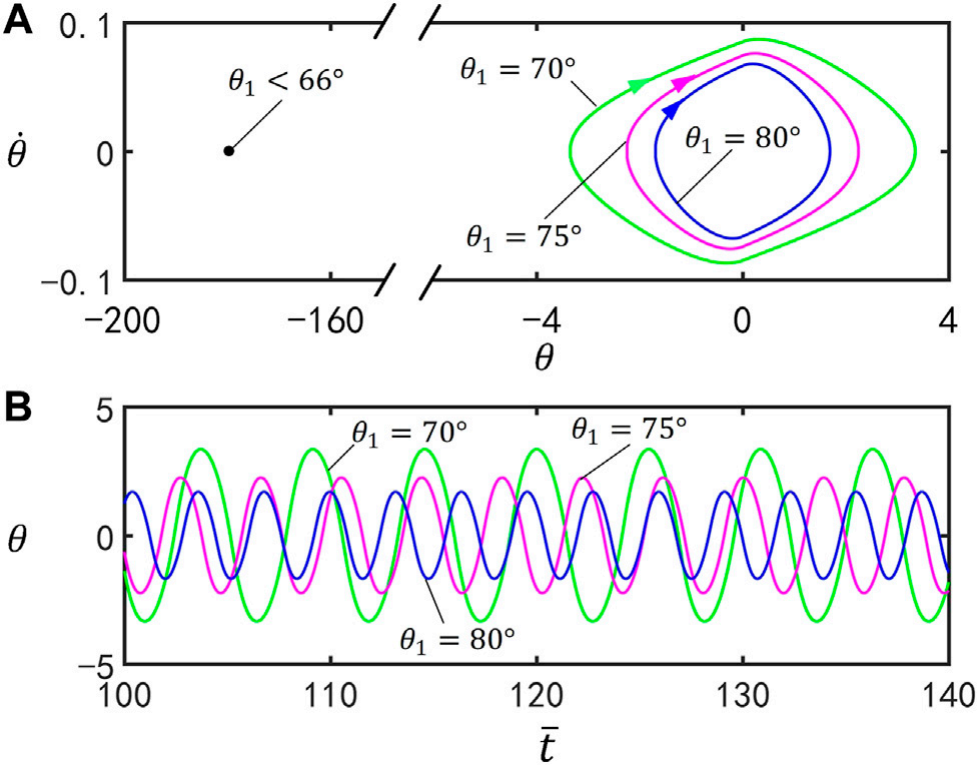
Effect of the vertex angle on self-stabilized oscillation of the inverted pendulum for $\bar{I}=0.25$ , $C_{0}=0.4$ , $\bar{g}=9.8$ , $\bar{\zeta }=14.7$ , $\theta_{0}=0{}^{\circ}$ , and ${\dot{\theta }}_{0}=0$ . **(A)** Limit cycles for $\theta_{1}=70{}^{\circ}$ $\theta_{1}=75{}^{\circ}$ , and $\theta_{1}=80{}^{\circ}$ . **(B)** Time series for $\theta_{1}=70{}^{\circ}$ $\theta_{1}=75{}^{\circ}$ , and $\theta_{1}=80{}^{\circ}$ . The amplitude and period of self-stabilized oscillation decreases with the increase of $\theta_{1}$ .

Figure 4 illustrates the effect of $\theta_{1}$ on self-stabilized oscillation of the inverted pendulum. In the computation, we set $\bar{I}=0.25$ , $C_{0}=0.4$ , $\bar{g}=9.8$ , $\bar{\zeta }=14.7$ , $\theta_{0}=0{}^{\circ}$ , and ${\dot{\theta }}_{0}=0$ . Figure 4A plots limit cycles for $\theta_{1}=70{}^{\circ}$ $\theta_{1}=75{}^{\circ}$ , and $\theta_{1}=80{}^{\circ}$ . The motion mode of the inverted pendulum can be changed by regulating the value of the parameters $\theta_{1}$ . Through calculation, for $\theta_{1}<66{}^{\circ}$ , the inverted pendulum eventually evolves into static mode at θ=180° , and for $\theta_{1}\geq 66{}^{\circ}$ , the inverted pendulum oscillates under steady illumination. This result means that there exists a critical vertex angle for the self-stabilized oscillation. This is because, for smaller $\theta_{1}$ , the V-shaped inverted pendulum is prone to be on the side of the *y*-axis, and the gravity moment is difficult to reverse. Figure 4B presents the time series for $\theta_{1}=70{}^{\circ}$ $\theta_{1}=75{}^{\circ}$ , and $\theta_{1}=80{}^{\circ}$ . It can be seen from Figures 4A,B that the amplitude and period of self-stabilized oscillation decreases with the increase of $\theta_{1}$ .

### B. Effect of the Initial Position

**FIGURE 5:**
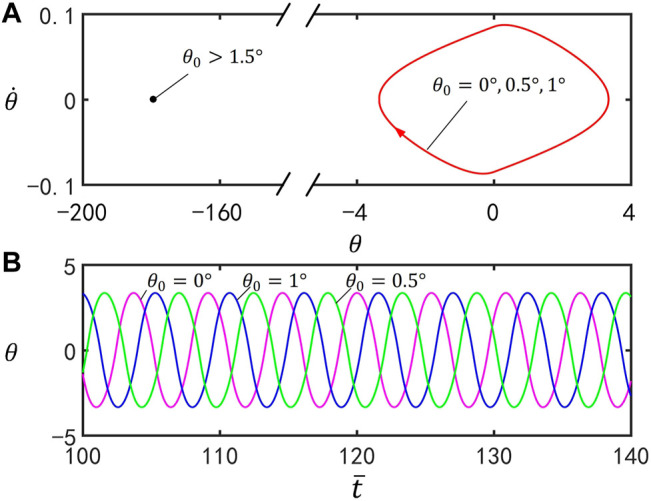
Effect of the initial position $\theta_{0}$ on self-stabilized oscillation of the inverted pendulum for $\bar{I}=0.25$ , $C_{0}=0.4$ , $\bar{g}=9.8$ , $\bar{\zeta }=14.7$ , $\theta_{1}=70{}^{\circ}$ , and ${\dot{\theta }}_{0}=0$ . **(A)** Limit cycles for $\theta_{0}=0{}^{\circ}$ , 0.5° , and 1° . **(B)** Time series curves for $\theta_{0}=0{}^{\circ}$ , 0.5° , and 1° . It can be seen that the initial position $\theta_{0}$ has no influence on the amplitude and period of self-stabilized oscillation.

Figure 5 delineates the effect of the initial position $\theta_{0}$ on self-stabilized oscillation of the inverted pendulum. In the computation, we set $\bar{I}=0.25$ , $C_{0}=0.4$ , $\bar{g}=9.8$ , $\bar{\zeta }=14.7$ , $\theta_{1}=70{}^{\circ}$ , and ${\dot{\theta }}_{0}=0$ . Figure 5A provides the limit cycles for $\theta_{0}=0{}^{\circ}$ , 0.5° , and 1° , and the three limit cycles are identical. Figure 5B plots the time series curves for $\theta_{0}=0{}^{\circ}$ , 0.5° , and 1° . By adjusting the value of parameter $\theta_{0}$ , the motion mode of the inverted pendulum can be found. Considering the symmetry of the structure, we only discuss the case of $\theta_{0}\geq 0{}^{\circ}$ . For $\theta_{0}\leq 1.5{}^{\circ}$ , the inverted pendulum oscillates under steady illumination and the amplitude does not change as the value of $\theta_{0}$ increases, whereas for $\theta_{0}>1.5{}^{\circ}$ , the LCE bar eventually evolves into static at θ=180° . It can be understood that, for large $\theta_{0}$ , the gravity moment in Eq. 6 cannot be reversed to bring the inverted pendulum back to the upper equilibrium position θ=0 .

### C. Effect of the Light Intensity

**FIGURE 6:**
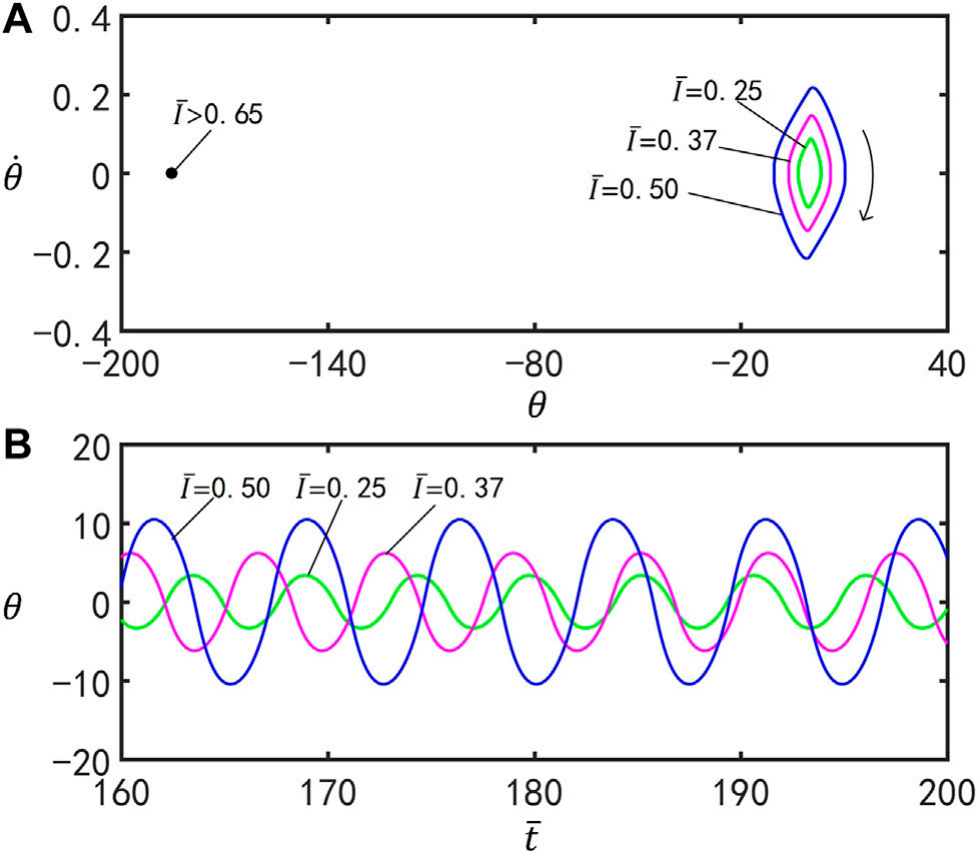
Effects of the light intensity $\bar{I}$ on self-stabilized oscillation of the inverted pendulum for $C_{0}=0.4$ , $\bar{g}=9.8$ , $\bar{\zeta }=14.7$ , $\theta_{0}=0{}^{\circ}$ , $\theta_{1}=70{}^{\circ}$ , and ${\dot{\theta }}_{0}=0$ . **(A)** Limit cycle for $\bar{I}=0.25$ , $\bar{I}=0.37$ , and $\bar{I}=0.50$ . **(B)** Time series for $\bar{I}=0.25$ , $\bar{I}=0.37$ , and $\bar{I}=0.50$ . The amplitude and period of the self-stabilized oscillation increase with the enhancement of $\bar{I}$ .

Figure 6 plots the effect of the dimensionless light intensity $\bar{I}$ on self-stabilized oscillation of the inverted pendulum. In the computation, we set $C_{0}=0.4$ , $\bar{g}=9.8$ , $\bar{\zeta }=14.7$ , $\theta_{0}=0{}^{\circ}$ , $\theta_{1}=70{}^{\circ}$ , and ${\dot{\theta }}_{0}=0$ . Figure 6A presents the corresponding limit cycle, and the time series curves for $\bar{I}=0.25$ , $\bar{I}=0.37$ , and $\bar{I}=0.50$ are shown in Figure 6B. The motion mode of the inverted pendulum can be changed by varying the value of the parameters within a certain range. For $\bar{I}>0.65$ , the inverted pendulum eventually evolves into static mode at θ=180° . For $\bar{I}\leq 0.65$ , the inverted pendulum oscillates. It can be seen from Figures 6A,B that the amplitude of the inverted pendulum increases with the growth of the dimensionless light intensity. This is because the increase of dimensionless light intensity motivates the maximum strain of the two bars of the inverted pendulum to augment, which greatly raises the gravity moment and expands the amplitude of the inverted pendulum.

### D. Effect of the Contraction Coefficient

**FIGURE 7:**
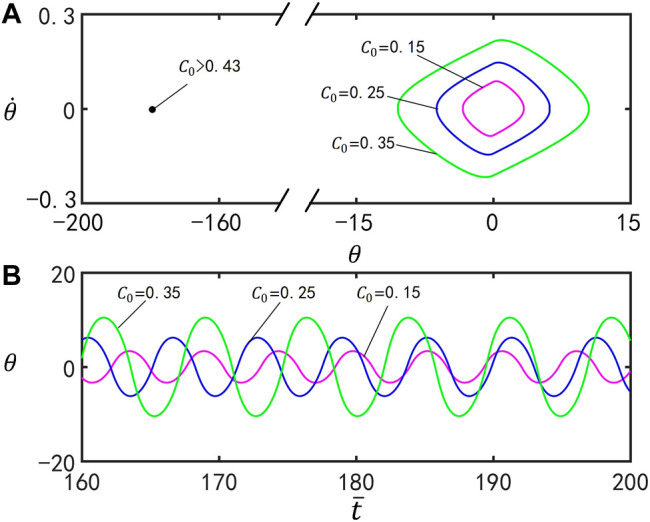
Effect of the contraction coefficient $C_{0}$ on self-stabilized oscillation of the inverted pendulum for $\bar{I}=0.6$ , $\bar{g}=9.8$ , $\bar{\zeta }=14.7$ , $\theta_{0}=0{}^{\circ}$ , $\theta_{1}=70{}^{\circ}$ , and ${\dot{\theta }}_{0}=0$ . **(A)** Limit cycles for $C_{0}=0.15$ , $C_{0}=0.25$ , and $C_{0}=0.35$ . **(B)** Time series for $C_{0}=0.15$ , $C_{0}=0.25$ , and $C_{0}=0.35$ . The amplitude and period of self-stabilized oscillation increase by increasing $C_{0}$ .

Figure 7 reflects the effect of the contraction coefficient $C_{0}$ on self-stabilized oscillation of the inverted pendulum. In the computation, we set $\bar{I}=0.6$ , $\bar{g}=9.8$ , $\bar{\zeta }=14.7$ , $\theta_{0}=0{}^{\circ}$ , $\theta_{1}=70{}^{\circ}$ , and ${\dot{\theta }}_{0}=0$ . Figure 7A plots the limit cycles for $C_{0}=0.15$ , $C_{0}=0.25$ , and $C_{0}=0.35$ . Figure 7B presents the time series for $C_{0}=0.15$ , $C_{0}=0.25$ , and $C_{0}=0.35$ . The motion mode of the inverted pendulum can be changed by tuning the value of the contraction coefficient. For $C_{0}>0.43$ , the inverted pendulum eventually evolves into static mode at θ=180° . For $C_{0}\leq 0.43$ , the inverted pendulum oscillates under steady illumination. With the increase of $C_{0}$ , the amplitude and period of the self-excited oscillation enlarge. This is because increasing contraction coefficient boosts the maximum strain of the two bars of the inverted pendulum, and then the gravity center of the inverted pendulum changes greatly, causing the gravity moment and, hence, the amplitude of the self-stabilized oscillation to rise.

### E. Effect of the Damping Coefficient

**FIGURE 8:**
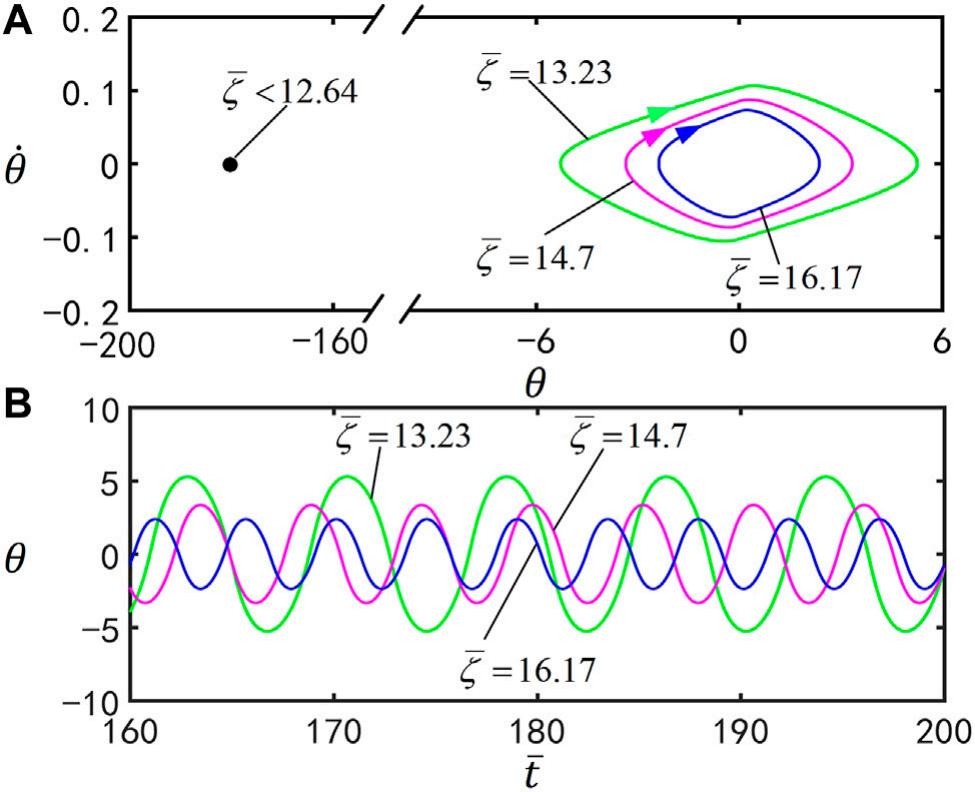
Effect of the damping coefficient $\bar{\zeta }$ on the self-stabilized oscillation of the inverted pendulum for $\bar{I}=0.25$ , $C_{0}=0.4$ , $\bar{g}=9.8$ , $\theta_{0}=0{}^{\circ}$ , $\theta_{1}=70{}^{\circ}$ , and ${\dot{\theta }}_{0}=0$ . **(A)** Limit cycle for $\bar{\zeta }=13.23$ , $\bar{\zeta }=14.7$ , and $\bar{\zeta }=16.17$ . **(B)** Time series for $\bar{\zeta }=13.23$ , $\bar{\zeta }=14.7$ , and $\bar{\zeta }=16.17$ . The amplitude and period of self-stabilized oscillation decrease by increasing $\bar{\zeta }$ .

Figure 8 illustrates the effect of the dimensionless damping coefficient $\bar{\zeta }$ on self-stabilized oscillation of the inverted pendulum. In the computation, we set $\bar{I}=0.25$ , $C_{0}=0.4$ , $\bar{g}=9.8$ , $\theta_{0}=0{}^{\circ}$ , $\theta_{1}=70{}^{\circ}$ , and ${\dot{\theta }}_{0}=0$ . Figure 8A presents the corresponding limit cycle, and the time series curves for $\bar{\zeta }=13.23$ , $\bar{\zeta }=14.7$ , and $\bar{\zeta }=16.17$ are shown in Figure 8B. By varying the parameter $\bar{\zeta }$ , the motion mode of the inverted pendulum can be adjusted. For $\bar{\zeta }<12.64$ , the inverted pendulum eventually evolves into static mode at θ=180° , whereas for $\bar{\zeta }\geq 12.64$ , the inverted pendulum oscillates under steady illumination. This is because, for smaller $\bar{\zeta }$ , the inverted rotates more quickly and the LCE bars do not have enough time to deform to reverse the total moment. With the increase of $\bar{\zeta }$ , the amplitude of the inverted pendulum decreases, which is consistent with the intuition.

### F. Effect of the Gravitational Acceleration

**FIGURE 9:**
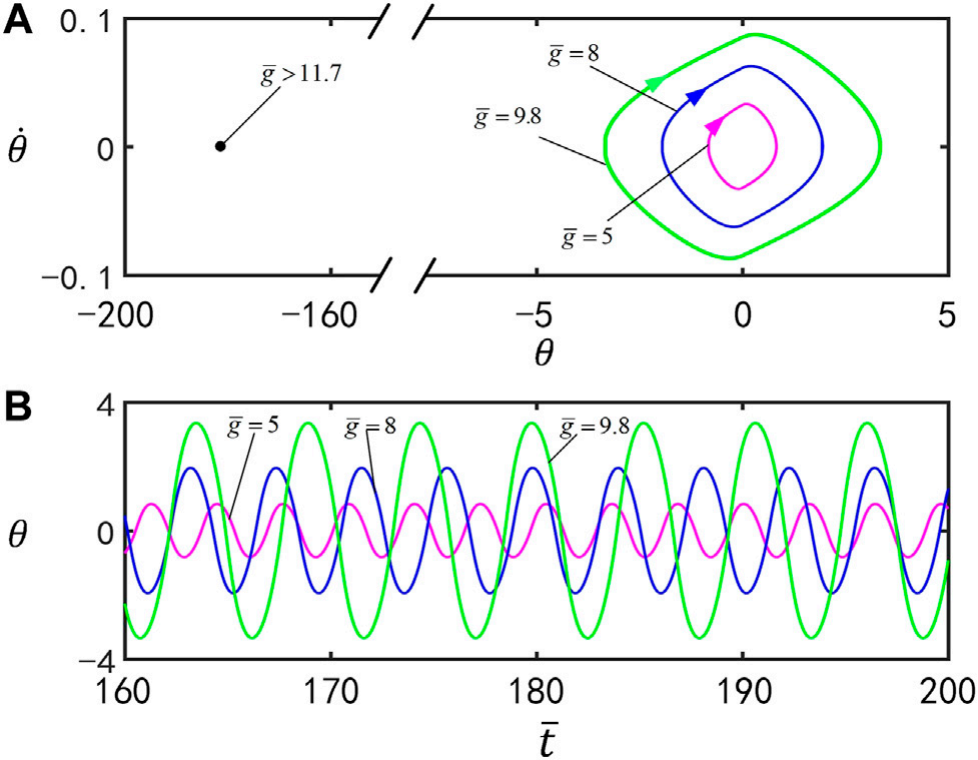
Effect of the gravitational acceleration $\bar{g}$ on self-stabilized oscillation of the inverted pendulum for $\bar{I}=0.25$ , $C_{0}=0.4$ , $\bar{\zeta }=14.7$ , $\theta_{0}=0{}^{\circ}$ , $\theta_{1}=70{}^{\circ}$ , and ${\dot{\theta }}_{0}=0$ . **(A)** Limit cycles for $\bar{g}=5$ , $\bar{g}=8$ , and $\bar{g}=9.8$ . **(B)** Time series for $\bar{g}=5$ , $\bar{g}=8$ , and $\bar{g}=9.8$ . The amplitude and period of self-stabilized oscillation increase by increasing $\bar{g}$ .

Figure 9 plots the effect of the dimensionless gravitational acceleration $\bar{g}$ on self-stabilized oscillation of the inverted pendulum. In the computation, we set $\bar{I}=0.25$ , $C_{0}=0.4$ , $\bar{\zeta }=14.7$ , $\theta_{0}=0{}^{\circ}$ , $\theta_{1}=70{}^{\circ}$ , and ${\dot{\theta }}_{0}=0$ . Figure 9A presents the limit cycles for $\bar{g}=5$ , $\bar{g}=8$ , and $\bar{g}=9.8$ . Figure 9B displays the time series for $\bar{g}=5$ , $\bar{g}=8$ , and $\bar{g}=9.8$ . The motion mode of the inverted pendulum can be changed by tuning the value of the gravitational acceleration. For $\bar{g}\leq 11.7$ , the inverted pendulum oscillates under steady illumination. With the increase of $\bar{g}$ , the amplitude and period of the self-stabilized oscillation rise. According to the physical meaning of $\bar{g}$ , the larger $\bar{g}$ is, the slower the *cis*-to-*trans* conversion is. Therefore, the smaller the total moment $M_{z}$ is, as shown in Eq. 6, the larger the amplitude and period are. For $\bar{g}>11.7$ , the inverted pendulum eventually evolves into static mode at θ=180° . This is because, for large $\bar{g}$ , the amplitude is large, and then the total moment $M_{z}$ is not large enough to bring the inverted pendulum back. The result may have its potential applications in aerospace or under a strong electromagnetic field.

The above results show that the increase of the light intensity, contraction coefficient, and gravitational acceleration, and the decrease of the vertex angle and damping coefficient are more conducive to the stability of the inverted pendulum system. Detailed numerical calculation shows that the optimal parameter combination is $\bar{I}=0.25$ , $C_{0}=0.4$ , $\bar{g}=9.8$ , $\bar{\zeta }=14.7$ , $\theta_{0}=0{}^{\circ}$ , $\theta_{1}=70{}^{\circ}$ , and ${\dot{\theta }}_{0}=0$ . This result has guiding significance for the design of inverted pendulums and related robots.

## V. Conclusion

In this paper, self-excited oscillation is adopted to propose a new self-stabilized inverted pendulum, which consists of a V-shaped optically responsive LCE bar. Based on the well-established dynamic LCE model, the nonlinear dynamic theory of the LCE inverted pendulum under steady illumination is formulated, and numerical calculation shows that the inverted pendulum system always evolves into the static state or the self-stabilized oscillation state. The mechanism of the self-stabilized oscillation is elucidated by the reversal of the gravity moment of the inverted pendulum. The amplitude and frequency of the self-oscillation can be designed by modulating several system parameters. The self-stabilized inverted pendulum fueled by steady illumination is sustainable and does not need an additional controller. The results provide new insights into understanding the self-oscillation phenomenon and offer new designs of inverted pendulum systems for potential applications in robotics, military industry, aerospace and, other fields.

## Data Availability

The original contributions presented in the study are included in the article/Supplementary Material, further inquiries can be directed to the corresponding author.
